# Sensitive Detection of SARS-CoV-2 Variants Using an Electrochemical Impedance Spectroscopy Based Aptasensor

**DOI:** 10.3390/ijms232113138

**Published:** 2022-10-28

**Authors:** Assem Kurmangali, Kanat Dukenbayev, Damira Kanayeva

**Affiliations:** 1 Department of Biology, School of Sciences and Humanities, Nazarbayev University, Astana 010000, Kazakhstan; 2 Department of Electrical and Computer Engineering, School of Engineering and Digital Sciences, Nazarbayev University, Astana 010000, Kazakhstan

**Keywords:** SARS-CoV-2, spike glycoprotein, virus, aptasensor, electrochemical impedance spectroscopy (EIS), aptamer

## Abstract

The global pandemic caused by severe acute respiratory syndrome coronavirus 2 (SARS-CoV-2) caused a threat to public health and a worldwide crisis. This raised the need for quick, effective, and sensitive detection tools to prevent the rapid transmission rate of the infection. Therefore, this study aimed to develop an electrochemical impedance spectroscopy (EIS)-based aptasensor employing an interdigitated gold electrode (IDE) to detect SARS-CoV-2 Spike (S) glycoprotein and viral particles. This allowed us to sensitively detect SARS-CoV-2 S glycoprotein with a limit of detection (LOD) of 0.4 pg/mL in a buffer solution and to obtain a linear increase for concentrations between 0.2 to 0.8 pg/mL with high specificity. The proposed aptasensor also showed a good sensitivity towards the heat-inactivated SARS-CoV-2 variants in a buffer solution, where the Delta, Wuhan, and Alpha variants were captured at a viral titer of 6.45 ± 0.16 × 10^3^ TCID_50_/mL, 6.20 × 10^4^ TCID_50_/mL, and 5.32 ± 0.13 × 10^2^ TCID_50_/mL, respectively. Furthermore, the detection of SARS-CoV-2 performed in a spiked human nasal fluid provided an LOD of 6.45 ± 0.16 × 10^3^ TCID_50_/mL for the Delta variant in a 50 µL sample and a detection time of less than 25 min. Atomic force microscopy images complemented the EIS results in this study, revealing that the surface roughness of the IDE after each modification step increased, which indicates that the target was successfully captured. This label-free EIS-based aptasensor has promising potential for the rapid detection of SARS-CoV-2 in complex clinical samples.

## 1. Introduction

The outbreak of the coronavirus disease (COVID-19) caused by the severe acute respiratory syndrome coronavirus 2 (SARS-CoV-2) that emerged in Wuhan province currently affects more than 100 countries. According to WHO, the number of affected people was 610 million people globally with approximately 6.5 million deaths [1]. In addition, the spread of the coronavirus disease and the restrictions, such as lockdowns, had an impact on the social and economic aspects of our lives, causing distress and uncertainty [2,3]. Another problem has been the rapid transmission rate of the infection. It is still of great concern as the number of people being infected is increasing. 

The SARS-CoV-2 virus belongs to the betacoronavirus group, has a positive single-stranded (ss) RNA genome and is structurally surrounded by four proteins known as the spike (S), envelope (E), matrix (M), and nucleocapsid (N) proteins [4]. Trimeric S glycoprotein exposed on the surface of the virus comprises the S1 and S2 subunits with 1273 amino acids in length. The S1 subunit plays an important role since it includes the receptor-binding domain (RBD) in the 319–542 region [5]. Various structural studies have demonstrated that the RBD of the SARS-CoV-2 S glycoprotein is responsible for the binding to the human angiotensin-converting enzyme 2 (ACE2), which initiates the viral entry into the host cells [6,7,8,9].

Currently, the SARS-CoV-2 virus is detected using real-time reverse-transcriptase polymerase chain reaction (RT-PCR), which is considered a gold standard and routinely used for the identification of the virus; however, there are several limitations related to this method. It is time-consuming and shows low sensitivity and accuracy. Besides, conducting this test requires the use of expensive equipment and trained personnel. Additionally, RT-PCR sometimes also produces false negative results [10]. Therefore, there is a need for fast, sensitive, and specific detection tools. Among them, biosensing devices are gaining interest due to their sensitivity and rapidness. To date, various biosensors have been developed and introduced to detect the SARS-CoV-2 S glycoprotein (Table 1). Several studies have reported the development of biosensors using antibodies as recognition agents that target the SARS-CoV-2 antigen [11,12,13]. Seo et al. [12] developed a field-effect transistor-based (FET) biosensing detector coated with graphene sheets and layered with a specific antibody against the S glycoprotein of SARS-CoV-2. The FET-biosensor could detect the SARS-CoV-2 antigen at the concentration of 1 fg/mL in phosphate-buffered saline (PBS) and 100 fg/mL concentration in the clinical transport medium. Another research group developed a label-free electrochemical immunoassay using a screen-printed electrode (SPE) functionalized with a monoclonal anti-spike antibody. The testing platform obtained a detection limit of 250 nM for S glycoprotein and 5.5 × 10^5^ PFU/mL for SARS-CoV-2 with a 45 min incubation time [13]. However, antibody-based sensors have several limitations that include stability issues, higher production costs of antibodies, and difficulty of chemical modifications [14]. Strip-based sensors that include lateral flow immunoassays (LFIA) are also being widely employed for the diagnosis of SARS-CoV-2, mainly for the detection of antibodies [15,16,17,18]. However, they cannot be used for the early detection of the virus and need to be used in combination with other tests since the IgM and IgG are only detectable two weeks after the start of infection [19]. 

One of the attractive recognition elements for biosensor development is short ss oligonucleotides (DNA or RNA) called aptamers. They are capable of binding to the target with high specificity and affinity [20]. This feature makes them an excellent recognition agent for the detection of different viral proteins [21,22,23] and genes [24,25,26]. Aptamers are much smaller compared with antibodies; therefore, more aptamers can be functionalized to the detection surface [27]. Aptamer-based biosensors or aptasensors can detect the viral markers in small quantities and differentiate the infected cells from uninfected ones [28]. Furthermore, lower cost, low immunogenicity and toxicity, shorter production time, and a wider spectrum of targets make them advantageous compared with antibodies [29,30]. Currently, there are various aptamer-based sensors for the detection of SARS-CoV-2; however, the majority of studies have been targeted at the detection of the S glycoprotein itself and not the SARS-CoV-2 virus [14,31,32,33,34].

Electrochemical impedance spectroscopy (EIS) is a well-known method that measures the impedance at a range of frequencies and allows the analysis of the electrochemical properties of an electrode and electrochemical processes occurring on its surface [35,36]. EIS is advantageous compared with other techniques in several respects. It allows label-free and sensitive detection as well as miniaturization ability [37]. Additionally, according to the literature, EIS is milder than other voltametric techniques as it provides conditions that do not affect the sample-modified surface [38,39]. The sensitivity of the impedimetric biosensors is also determined by the electrode geometry and parameters such as the electrode gap width that increase the sensitivity of the sensor [40]. For these reasons, there is substantial interest in the use of interdigitated electrodes (IDEs), particularly as there is no need for additional electrodes, and the electrochemical setup is incorporated on the surface. An additional advantage is an ability to rapidly reach steady-state and easy miniaturization of the electrochemical system [41].

The recent spread of the SARS-CoV-2 virus suggests that there is a need for rapid detection techniques. Recently, Song et al. [42] selected aptamers that have high binding affinity and specificity towards the RBD region of the SARS-CoV-2 S glycoprotein using the ACE2 competition-based aptamer selection strategy. According to their data, the aptamers, CoV2-RBD-1C and CoV2-RBD-4C, demonstrated higher binding affinity towards the RBD domain with *K*_d_ values of 5.8 nM and 19.9 nM, respectively [42]. In this work, we developed the EIS-based aptasensor using the CoV2-RBD-1C [42] aptamer with the addition of a spacer 18-HEG (hexaethylene glycol) and 5 thymine residues that were fabricated on the surface of an IDE to detect the RBD of the SARS-CoV-2 S glycoprotein. The aptasensor does not require labeling and can detect the target within 25 min. The developed aptasensor was incubated with different concentrations of the S glycoprotein showing an LOD of 0.4 pg/mL. The aptasensor sensitivity was further tested on three variants of heat-inactivated SARS-CoV-2 with a detection limit of 6.45 ± 0.16 × 10^3^ TCID_50_/mL (Delta), 6.20 × 10^4^ TCID_50_/mL (Wuhan), and 5.32 ± 0.13 × 10^2^ TCID_50_/mL (Alpha) in 50 µL samples. The surface characterization of each modification step using atomic force microscopy (AFM) confirmed the immobilization and functionalization of the IDE surface. Furthermore, the performance of the aptasensor was studied to detect the heat-inactivated Delta variant spiked in a nasal fluid to mimic the clinical environment, obtaining a sensitivity of 6.45 ± 0.16 × 10^3^ TCID_50_/mL. 

**Table 1:** Comparative table of recent biosensors for the detection of SARS-CoV-2 S glycoprotein.

Detection Method	Transducer	Recognition Agent	Protein Incubation and Detection Time	Detection Range	LOD	References
FET	Graphene sheet	SARS-CoV-2 Antibody	Real time	1 fg/mL–10 pg/mL 1.6 × 10^1^–1.6 × 10^4^ pfu/mL	1 fg/mL (SARS-CoV-2 antigen); 1.6 × 10^1^ pfu/mL (SARS-CoV-2); 2.42 × 10^2^ copies/mL in clinical samples	[12]
Electrochemical	Co-TNTs	-	30 s detection time	14–1400 nM	0.7 nM	[43]
Electrochemical	Graphene functionalized with PBASE linker	SARS-CoV-2 antibody	45 min incubation time	260–1040 nM 34.38 × 10^3^ to 5.50 × 10^5^ PFU/mL	260 nM (20 μg/mL) S1 glycoprotein; 5.5 × 10^5^ PFU/mL (SARS-CoV-2)	[13]
Electrochemical	SPCE modified with Cu_2_O NCs	Recombinant anti-SARS-CoV-2 S antibody	20 min	0.25 fg mL–1 μg mL	0.04 fg mL^−1^	[44]
Electrochemical	Chitosan/CdS QDs-gC_3_N_4_/ITO electrode	CoV2-RBD-1C DNA aptamer	40 min incubation time	0.5–32.0 nM	0.12 nM	[32]
Electrochemical	GPE + AuNPs	ACE-2 receptor	6.5 min detection including 5 min incubation time	1 × 10^−12^ to 1 × 10^−10^ g·mL ^−1^	229 fg·mL^−1^	[45]
Electrochemical	SPCE + AuNPs	CoV2-RBD-1C DNA aptamer Thiol C6 S-S modification (5/ ThioMC6-D/)	40 min incubation	10 pM–100 nM	1.30 pM (66 pg/mL)	[33]
SERS	Au nanopopcorn	CoV2-RBD-1C DNA aptamer	15 min	0–1000 PFU/mL of SARS-CoV-2	>10 PFU/mL	[46]
SERS	AgNPs	CoV2-RBD-1C DNA aptamer	7 min detection	5.5 × 10^4^ to 1.4 × 10^6^ TCID_50_/mL of SARS-CoV-2	5.5 × 10^4^ TCID_50_/mL	[47]
PIFE	Cy3	DNA aptamer	2 min detection	-	5.8 × 10^4^ TCID_50_ mL^−1^	[48]
SPR-POF	Au nanofilm modified with PEGthiol + BiotinPEGlipo + SA	Biotinylated CoV2-RBD-1C DNA aptamer	10 min incubation time	25–1000 nM	37 nM	[34]
Electrochemical	IDE	Thiolated CoV2-RBD-1C DNA aptamer with an 18-HEG spacer and thymine residues	2.5 min detection and 20 min incubation time	0.1–12.8 pg/mL of S glycoprotein; 10^1^ to 10^5^ TCID_50_/mL of SARS-CoV-2 variants (Alpha, Wuhan, and Delta)	0.4 pg/mL (S glycoprotein), 6.45 ± 0.16 × 10^3^ TCID_50_/mL (Delta), 6.20 × 10^4^ TCID_50_/mL (Wuhan), and 5.32 ± 0.13 × 10^2^ TCID_50_/mL (Alpha) in 0.01 M PBS. 6.45 ± 0.16 × 10^3^ TCID_50_/mL (Delta) spiked in human nasal fluid	This study

FET—field effect transistor; Co-TNT—cobalt-functionalized TiO_2_ nanotube; PBASE—1-pyrene butyric acid N-hydroxysuccinimide ester; NC—nanocube; CdS QD—cadmium sulfide quantum dot; gC_3_N_4_—graphitic carbon nitride; ITO—indium tin oxide; SERS—surface-enhanced Raman scattering; PIFE—protein-induced fluorescence enhancement; GPE—graphite pencil electrode; AuNP—gold nanoparticle; SPCE—screen-printed carbon electrode; PEG—polyethyleneglycol; BiotinPEGlipo-Biotin-dPEG^®^3-Lipoamide; SPR—surface plasmon resonance; POF—plastic optical fiber; IDE—interdigitated gold electrode.

## 2. Results and Discussions

### 2.1. Sensitivity and Specificity of the Aptasensor

**Scheme 1:**
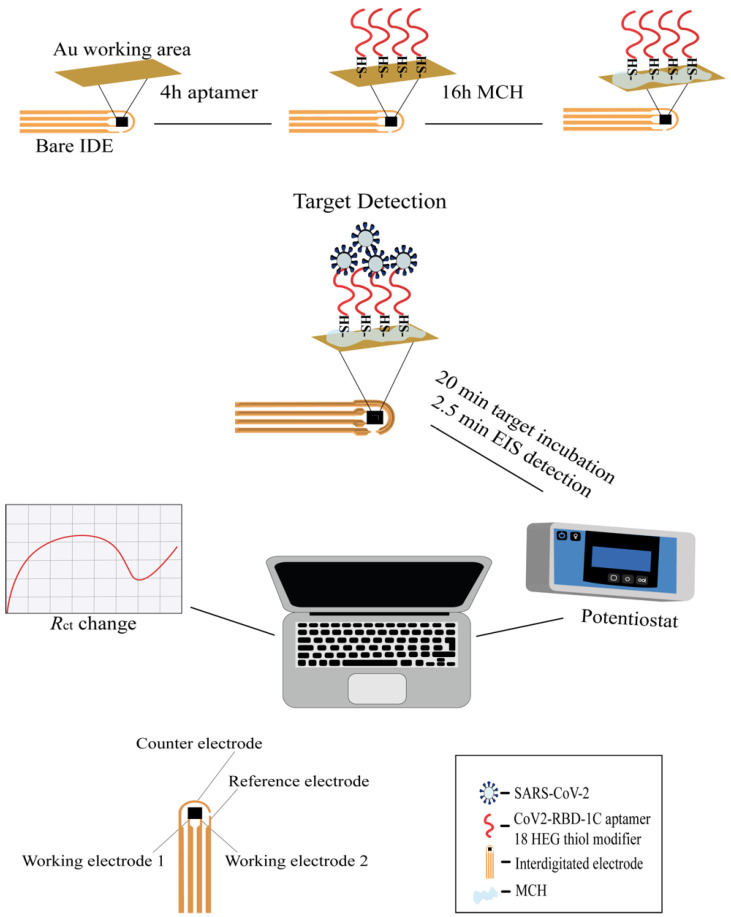
Workflow of the IDE surface functionalization and SARS-CoV-2 detection with the EIS aptasensor. The working surface of the IDE was functionalized with an aptamer (shown in red) and a 3 mM MCH. A 1 µM thiolated aptamer with an 18 HEG modifier was immobilized onto the IDE surface for 4 h followed by incubation with MCH for 16 h. After functionalization, the IDE was exposed to different concentrations of the target protein or SARS-CoV-2 variants for 20 min, and the signal measurements were recorded by the PSTrace 5.8 software using a portable potentiostat that was connected to the IDE through a cable connector. The IDE was composed of two working electrodes (WE), an auxiliary electrode, and a reference electrode. The WEs were composed of 52 pairs (bands), with a band gap and width of 10 μm, and a surface area of 4 mm^2^.

EIS has emerged as an effective and powerful technique for the study of the electrical properties occurring on the interface between the electrode and the immobilized aqueous solution. Impedimetric measurements are performed by applying the potential to the working electrode and measuring the changes in current, impedance, and charge transfer resistance after the binding of the target [37]. In this study, the thiolated aptamers were immobilized onto the IDE working surface. The workflow of the study is presented on Scheme 1. The sensitivity of the aptasensor was tested by measuring the different concentrations of the RBD S glycoprotein ranging from 0.1 pg/mL to 12.8 pg/mL with a 20 min incubation. The choice of the aptamer sequence was based on a previous study [42], where the aptamers with high binding affinity were selected towards the RBD region of SARS-CoV-2 S glycoprotein. We functionalized the aptamer sequence at the 5′-terminus with the addition of a thiol (-SH) C6 group and internal modifications of an 18-HEG spacer and 5-T residues. Internal modifications were included to create spacing between the aptamer sequence and the IDE surface and, therefore, ensure a better interaction with the target due to the upright orientation of the aptamer on the surface [49,50]. In addition, HEG residues (-OCH_2_CH_2_-) help to minimize steric hindrance, reduce the non-specific binding, and are considered to be sequence-neutral [51,52]. Thymine bases in the aptamer sequence were preferred over other nucleotides due to their ability to adsorb less strongly on a gold surface [53]. After aptamer immobilization, IDE was incubated with MCH which is commonly used for impedimetric measurements since it acts as a co-immobilizing agent that reduces non-specific interactions between the aptamer and gold surface and also provides a better orientation for the aptamer [54,55]. The final concentration of the modified aptamer used in this study was 1 μM based on the protocol developed by [56]; however, further research should be conducted to investigate the optimal aptamer density. This is important as the densely packed surface might prevent the effective binding of the protein to the aptamer [49].

**Figure 1:**
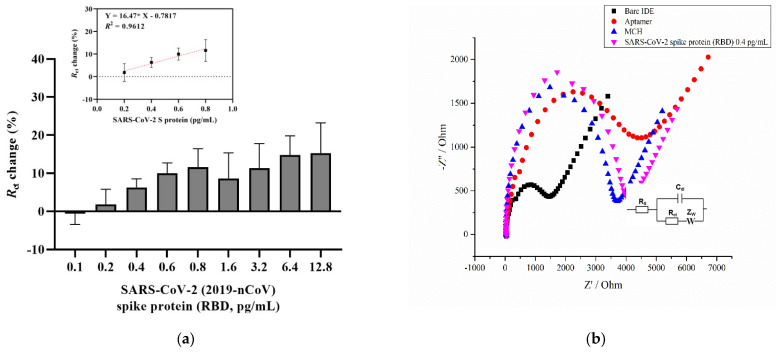
(**a**) A concentration-dependent curve for SARS-CoV-2 S glycoprotein detection using an aptamer and MCH immobilization steps relative to blank measurements from the EIS signal. The inset shows the calibration curve from 0.2 to 0.8 pg/mL linear increase. The EIS measurements were recorded in a 2 mM ferro/ferricyanide [Fe(CN)_6_]^3−^/^4−^ redox couple. Error bars show the mean for three measurements from three different electrodes. (**b**) The representative Nyquist plot (-Z′ vs. Z′) of the bare IDE after a 4 h aptamer, a 16 h 3 mM MCH immobilization, and a 0.4 pg/mL SARS-CoV-2 S glycoprotein detection in the frequency range 0.1 Hz to 50 kHz with a 10 mV a.c. Inset: Randles equivalent circuit, where *R*_s_ is the solution resistance, *R*_ct_ is the charge transfer resistance, *C*_dl_ is the double layer capacitance, and *Z*_w_ is the Warburg element.

Figure 1a shows a concentration-dependent curve which shows an increasing trend in the charge transfer resistance percentage with increasing analyte concentration. The aptasensor results showed a linear increase for the concentrations between 0.2 and 0.8 pg/mL (Figure 1a, inset). The aptasensor results showed a linear regression, with an equation of *y* = 0.01647 × *x* − 0.7817, and a correlation coefficient of *R*^2^ = 0.9621 (* *p* < 0.05), where *x* is the concentration of the RBD S glycoprotein. The Randles equivalent circuit was used to fit the experimental data that included *R*_s,_ which is the solution resistance, the charge transfer resistance (*R*_ct_), the double layer capacitance (*C*_dl_), and the Warburg impedance (*Z*_w_) (Figure 1b, inset). Figure 1b provides a representative Nyquist plot of each functionalization step that consists of a semicircle at high frequencies indicating the charge-transfer resistance and a vertical linear region that appears at the lower frequency ranges indicating diffusion resistance [57]. As can be seen, the cleaned bare surface of the IDE showed a small semicircle (*R*_ct_ = 1191 Ω) compared with the diameter of the other semicircles. This indicates the rapid electron transfer of the ferri/ferrocyanide ions (Fe(CN)_6_]^3−/4−^) to the bare IDE surface. Upon immobilization of the aptamer and further overnight incubation with a blocking solution (MCH), the charge transfer resistance gradually increased to 3236 Ω and 3385.1 Ω, respectively. An increase in charge transfer resistance after immobilization of the aptamers indicates that negative electroactive ions of the redox buffer repel the negatively charged phosphate groups of the DNA aptamer, which confirms its attachment to the surface [35,44,58]. Finally, after 20 min incubation with 0.4 pg/mL of the target protein, the *R*_ct_ increased to 3727 Ω. In the current study, the LOD for the SARS-CoV-2 S glycoprotein was found to be 0.4 pg/mL, which was calculated using the formula LOD = 3 × (*S*_a_/b), where *S*_a_ is the standard deviation of the y-intercept, and *b* is the slope of the calibration curve [59]. This result, in combination with other findings, provides a contribution to the detection of the SARS-CoV-2 S glycoprotein (Table 1).

**Figure 2:**
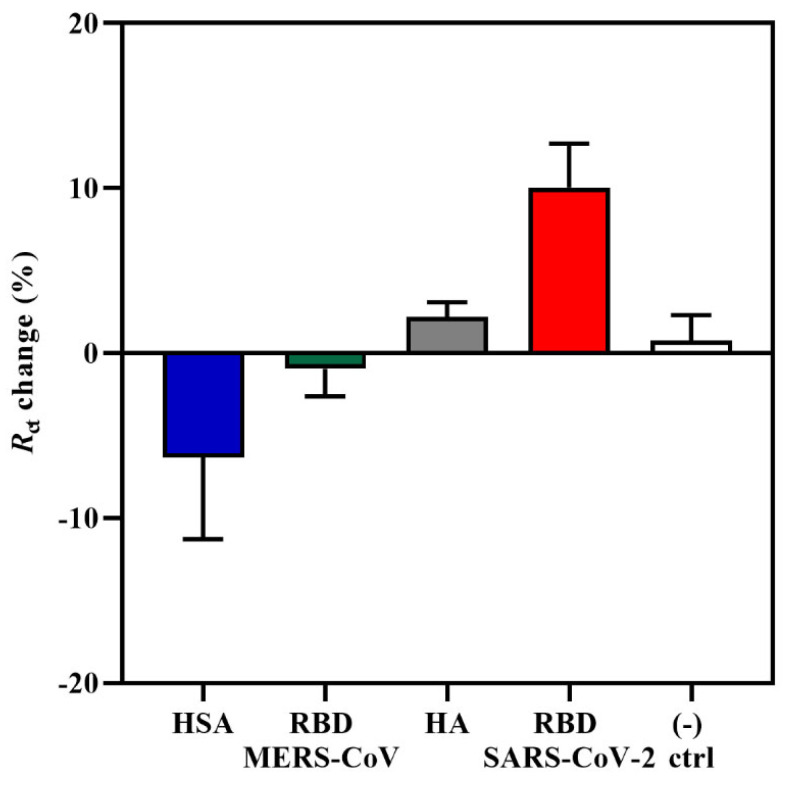
An EIS specificity response towards 0.6 pg/mL of MERS-CoV RBD S glycoprotein, influenza HA peptide, HSA, and the target SARS-CoV-2 S glycoprotein. A buffer without the aptamer was used as a negative control. Data are presented as the means of ± standard deviation and standard error of the mean (*n* = 3).

The specificity of the aptasensor was tested against three non-target proteins, such as the RBD domain of Middle East Respiratory Syndrome coronavirus (MERS-CoV) S glycoprotein, influenza hemagglutinin (HA) peptide, and human serum albumin (HSA) at a concentration of 0.6 pg/mL. This analysis was important for determining whether the increase in *R*_ct_ response and change in the impedance spectra was due to the specific interaction between the CoV2-RBD-1C aptamer and the RBD S glycoprotein and not due to non-specific bindings. MERS-CoV and SARS-CoV-2 come from the same betacoronavirus genus; therefore, both have the S glycoprotein that plays a significant role in the viral pathogenesis [60]. HA is a surface glycoprotein of the influenza virus, which is responsible for recognizing the host receptor, and given the similarity of symptoms between COVID-19 and influenza [61], we tested HA in this specificity study. HSA is the most abundant protein in human plasma and is considered an important biomarker of many diseases [62]. The aptasensor was incubated with target and non-target proteins for 20 min, and further, the response was measured. As can be seen in Figure 2, non-target proteins resulted in the lowest responses in comparison with SARS-CoV-2 RBD S glycoprotein, showing a charge transfer resistance of −0.96%, 2.1%, and −6.32% for MERS S glycoprotein, HA, and HSA, respectively. The obtained responses from non-target proteins confirm the specificity of the aptasensor to the SARS-CoV-2 RBD S glycoprotein. Statistical analysis of the binding of the aptamer to the target and non-target proteins was performed by applying a one-way ANOVA with multiple comparisons (post hoc Tukey’s test), which showed that a significant difference was only observed between the RBD and HSA responses. Moreover, there was no increase in response for the negative control sample, where the IDE was incubated with the target protein that was previously functionalized for 4 h with only 0.01 M PBS (pH 7.4) and no aptamer on the surface. The signal showed an *R*_ct_ change response of only 0.76%. The results of the current study support the findings of [33], where the aptasensor response towards non-target MERS-CoV S protein showed an *R*_ct_ percentage change of only 7%, which is also lower compared with the sensitivity towards the target SARS-CoV-2 S glycoprotein.

### 2.2. Detection of Inactivated SARS-CoV-2 Variants

To further validate the sensitivity of the aptasensor, three variants of the heat-inactivated SARS-CoV-2 in a detection range of 10^1^ to 10^5^ TCID_50_/mL were analyzed. The Wuhan and Alpha variants were isolated from the nasopharyngeal swabs of patients with COVID-19 in Almaty, Kazakhstan, and the Delta variant of SARS-CoV-2 was isolated from a patient in the Mangystau region of Kazakhstan [63]. Ten-fold dilutions in 0.01 M PBS (pH 7.4) were prepared from the viral stock with the titer of 6.20 ± 0.00 log_10_ TCID_50_/mL (Wuhan), 5.32 ± 0.13 log_10_ TCID_50_/mL (Alpha), and 6.45 ± 0.16 log_10_ TCID_50_/mL (Delta). The infectious titer of SARS-CoV-2 variants was analyzed by evaluating the cytopathic effect of virus isolates and subsequently determining the 50% tissue culture infectious dose (TCID_50_/mL), which was calculated according to the Reed and Muench method (Tables S1 and S2) [64]. Cycle threshold (Ct) values from real-time RT-PCR with different concentrations of SARS-CoV-2 variants are presented in the Supplementary Material (Table S3).

**Figure 3:**
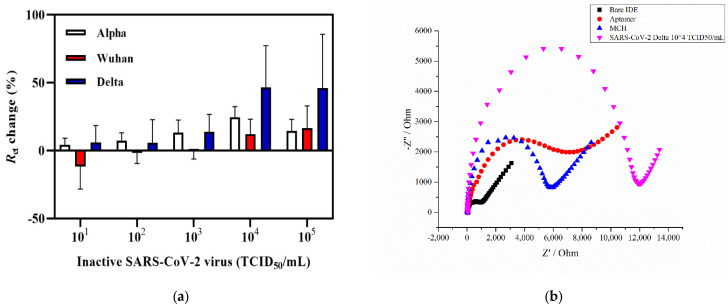
Detection of SARS-CoV-2 viral lysates. (**a**) Detection of three SARS-CoV-2 (Alpha, Delta, and Wuhan) variants in the concentration range of 10^1^ to 10^5^ TCID_50_/mL. (**b**) The representative Nyquist plot of a bare IDE, an aptamer functionalized, with an MCH backfilling, and capturing of 6.45 ± 0.16 × 10^4^ TCID_50_/mL of the Delta variant. Error bars show the mean of ± standard deviation and standard error (*n* = 3) for three measurements from 3 electrodes.

Figure 3a provides the graph of the *R*_ct_ change response of the electrochemical impedance aptasensor incubated with increasing concentrations of Alpha, Delta, and Wuhan variants of SARS-CoV-2. As shown in Figure 3a, incubation with the Delta variant of SARS-CoV-2 showed no response towards the first concentrations of 10^1^ and 10^2^ TCID_50_/mL, whereas further incubation with higher concentrations led to an increase in the signal, showing *R*_ct_ changes of 13.8%, 46.7%, and 46.2% for 10^3^, 10^4^, and 10^5^ TCID_50_/mL, respectively. As for the Wuhan variant of SARS-CoV-2, the lowest titer level that showed the sensor signal increase was 10^4^ and 10^5^ TCID_50_/mL with the charge transfer resistance change equaling 12.1% and 16.5%, respectively (Figure 3a). The coronavirus Alpha variant showed a response at 10^2^ TCID_50_/mL with an *R*_ct_ change % equal to 7.3% and increased to 24.6% at 10^4^ TCID_50_/mL. A representative Nyquist plot of each functionalized step is shown in Figure 3b. The *R*_ct_ of the bare IDE surface was 819 Ω, which then gradually increased with the aptamer immobilization and MCH blocking to 6158 Ω and 5548 Ω, respectively. The *R*_ct_ value also increased in the presence of 10^4^ TCID_50_/mL of SARS-CoV-2 Delta variant (*R*_ct_ = 10,534 Ω). A statistical analysis was performed by applying a two-way ANOVA with multiple comparisons, which showed that there was a statistically significant difference between the Wuhan and Delta variants (*n* = 3). The Alpha variant, also known as B.1.1.7, first emerged in the UK and was considered as a more transmissible strain with a new N501Y mutation at the 501 position of the RBD S glycoprotein replacing asparagine (N) with tyrosine (Y) [65]. In addition, the presence of the P681H mutation also contributes to the increased infection ability [66]. Both the Alpha and Delta variants have a similar enhanced transmission speed with the latter having major mutations in L452R that lead to a stronger binding affinity of the protein to the ACE-2 [67]. Compared with these two variants, the Wuhan variant carries fewer mutations, including D614G that does not affect the binding affinity of S glycoprotein to ACE-2 but increases the amount of functional S protein on the virus surface [68]. According to the obtained results, the detection limit for the Delta variant was estimated to be 6.45 ± 0.16 × 10^3^ TCID_50_/mL. It was 6.20 × 10^4^ TCID_50_/mL for the Wuhan variant, and 5.32 ± 0.13 × 10^2^ TCID_50_/mL for the Alpha variant in a 50 µL sample (Figure 3). A relatively better *R*_ct_ change (%) response was obtained for the Alpha and Delta variants, which could potentially indicate that the mutations present on the S glycoprotein do not affect the aptamer binding and the response since the CoV-RBD-1C was selected towards the RBD domain of wild-type SARS-CoV-2 virus [8,42,69]. According to the molecular docking simulations performed by [42], the selected aptamer forms hydrogen bonds with the RBD of the SARS-CoV-2 glycoprotein at Thr500, Gln506, and Asn437 positions, which leads to the binding between the aptamer and the target. These results are consistent with or have an improvement in the detection level of SARS-CoV-2 (Alpha variant) compared with that of previously reported studies in which an inactivated SARS-CoV-2 viral lysate was detected using a PIFE-based aptasensor with an LOD of 5.8 × 10^4^ infectious doses (TCID_50_/mL) [48]. Similarly, a SERS-based aptasensor developed by [47] detected a SARS-CoV-2 isolate with an LOD of 5.5 × 10^4^ TCID_50_/mL. In addition, in a parallel study based on a sandwich enzyme-linked oligonucleotide assay (ELONA) conducted by our research group [70], the SARS-CoV-2 inactivated variants in the buffer were detected in a viral titer from 6.20 × 10^2^ TCID_50_/mL for the Wuhan variant, 5.32 × 10^2^ TCID_50_/mL for the Alpha variant, and 6.45 × 10^2^ TCID_50_/mL for the Delta variant. The Delta variant was selected for subsequent spiking in nasal fluid due to the higher percentage *R*_ct_ change with an increase of up to 46.7% compared with the other two variants (Figure 3a).

### 2.3. IDE Surface Characterization

**Figure 4:**
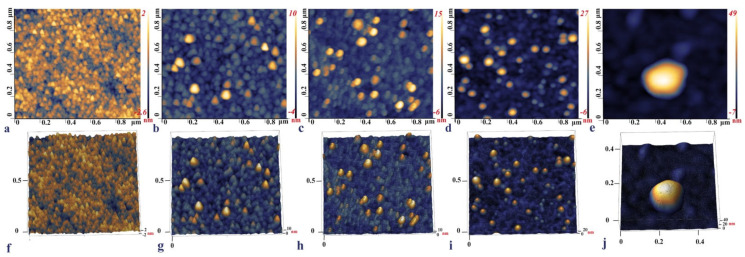
2D and 3D AFM images of the bare IDE surface after cleaning (**a**,**f**), IDE gold surface after 4 h incubation with the aptamer (**b**,**g**), 16 h MCH backfilling (**c**,**h**), and the aptamer functionalized IDE incubated with 0.6 pg/mL of the RBD S glycoprotein (**d**,**i**), and a 10^3^ TCID_50_/mL SARS-CoV-2 Delta variant captured on the aptamer functionalized IDE surface (**e**,**j**).

The surface topography of each IDE surface modification step was observed with the AFM, namely, the bare IDE, the aptamer functionalized, backfilled with MCH, with captured SARS-CoV-2 S glycoprotein, and a heat-inactivated SARS-CoV-2 Delta variant. (Figure 4a,f) shows that the bare IDE electrode has a grainy layer with a surface height of around 2–2.6 nm. This is in line with the published data, where the size of the bare Au surface was between 1.11 ± 0.03–4.9 nm and was characterized by a similar grainy surface [71,72,73]. Upon functionalization with the aptamer, the surface roughness increased up to 16.2 nm (Figure 4b,g). In the final step of incubation with the RBD S glycoprotein, the micrographs of the electrode with protein demonstrated a visible increase in height from 27 nm up to 34 nm (Figure 4d,i). These topography images of RBD S glycoprotein are consistent with those reported by [33], where binding of SARS-CoV-2 S glycoprotein and ssDNA aptamer with gold nanoparticles immobilized on SPCEs (screen-printed carbon electrodes) was analyzed.

The AFM imaging of the aptamer functionalized IDE surface capturing the Delta variant demonstrated that the inactivated viral particles had a round or close to the ellipsoidal shape with an individual particle size of around 56.6 nm (Figure 4e,j). Previous studies reported that the size of the SARS-CoV-2 ranged from 45 to 140 nm; however, the inactivation of the virus led to the deformation of the shape and size alterations [74]. The shrinkage of the shape and smaller sizes of the viral particle observed on the AFM micrograph could be due to the inactivation of the samples by heating to 65 °C for 30 min.

### 2.4. Detection of Inactivated SARS-CoV-2 Delta Variant in Human Nasal Fluid

**Figure 5:**
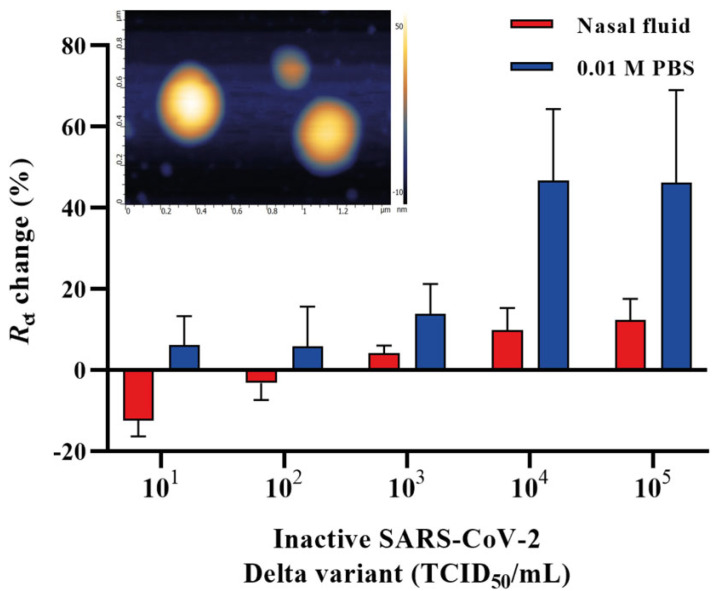
A concentration-dependent curve for the SARS-CoV-2 Delta variant spiked in nasal fluid and 0.01 M PBS (pH 7.4) detected by the EIS aptasensor. Inset: the AFM micrograph of SARS-CoV-2 Delta variant on a mica surface. Error bars show the mean of ± standard deviation and standard error (*n* = 3) for three measurements from 3 electrodes.

It is important to validate the feasibility of the developed aptasensor in a clinical environment. Therefore, we aimed to detect the heat-inactivated SARS-CoV-2 Delta variant by spiking it in a commercially available nasal fluid in the concentration range of 10^1^–10^5^ TCID_50_/mL and further applied it onto the aptamer functionalized IDE surface. The nasal fluid was diluted in 0.01 M PBS (pH 7.4) in a ratio of 1:1000, and different concentrations of the Delta variant were spiked in the nasal fluid. A 50 μL sample of each concentration was applied to the surface of aptamer functionalized IDE and incubated for 20 min. All electrodes were rinsed in 0.01 M PBS (pH 7.4) after the incubation to prevent non-specific interactions, and the signals were measured in redox couple buffer. Figure 5 shows the *R*_ct_ change response for the heat-inactivated SARS-CoV-2 Delta variant diluted in a buffer (0.01 M PBS, pH 7.4) and nasal fluid (1:1000). The detection of the Delta variant in the nasal fluid using the EIS aptasensor showed a sensitivity of 6.45 ± 0.16 × 10^3^ TCID_50_/mL.

The surface roughness of the inactivated SARS-CoV-2 Delta variant on a mica surface, used as a reference, demonstrated a size distribution ranging from 49 nm to 73.7 nm. The viral particles had round or spherical morphology when observed using the AFM (Figure 5, inset). An additional transmission electron microscopy (TEM) study also showed that the inactivated Delta variant of SARS-CoV-2 deposited on a carbon grid with a magnification of 50 nm and 100 nm (Figure S1) had a similar size distribution as that of in Figure 5 inset. Human nasal fluid is made up of mucin, which comprises 2% of the fluid, various lipids, abundant proteins such as lysozyme, lactotransferrin, immunoglobulins and albumin, enzymes and mediators such as kallikrein and kinins [75]. This could potentially interfere with the detection of the virus in such an environment and affect the response and, therefore, might result in a decrease in *R*_ct_ change % of the spiked samples. Another explanation for the low response might be that the adsorption of the nasal fluid to the electrode surface raised the background signal. Despite this, our developed aptasensor was able to detect the heat-inactivated SARS-CoV-2 Delta variant in the nasal fluid with an LOD of 6.45 ± 0.16 × 10^3^ TCID_50_/mL, requiring an overall 25 min detection time and demonstrating the great potential of the aptasensor for future work with patient samples.

## 3. Materials and Methods

### 3.1. Materials

CoV2-RBD-1C aptamer sequence 5′-CAGCACCGACCTTGTGCTTTGGGAGTGCTGGTCCAAGGGCGTTAATGGACA-3′ [42] was modified at the 5′-terminus with the addition of a thiol (-SH) C6 group, an 18-HEG spacer, and 5-T residues that were synthesized by Eurogentec (Seraing, Belgium). SARS-CoV-2 (2019-nCoV) Spike RBD recombinant protein (25.1 kDa; cat. no. 40592-VNAH; YP_009724390; Arg319-Phe541) and MERS-CoV Spike/RBD protein fragment (aa 367-606, His Tag; 27.7 kDa; cat. no. 40071-V08B1) were obtained from Sino Biological (Beijing, China). Influenza hemagglutinin (HA) peptide (cat. no. I2149), potassium hexacyanoferrate (III) (cat. no. 244023), potassium hexacyanoferrate (II) trihydrate (cat. no. P3289), 6-mercapto-1-hexanol (MCH) (cat. no. 725226), phosphate-buffered saline (PBS, 0.01 M, pH 7.4, cat. no. P4417), and tris (2-carboxyethyl) phosphine hydrochloride (TCEP, cat. no. C4706-2G) were purchased from Sigma-Aldrich (St. Louis, MO, USA). Human serum albumin (HSA) was purchased from Abcam (cat. no. ab205808, Cambridge, UK). Single donor human nasal fluid was purchased from Innovative Research (cat.no. IRHUNFSVIAL, Novi, MI, USA). All aqueous solutions were prepared using ultrapure water (18.2 MΩ cm, Thermo Scientific, Barnstead Smart2Pure, Waltham, MA, USA).

### 3.2. SARS-CoV-2 Variants

Three heat-inactivated virus isolates (BSL-1) grown in Vero E6 cell lines (i) hCoV-19/Kazakhstan/KazNAU-NSCEDI-4635/2020 (GISAID accession no. EPI_ISL_1208952; belongs to the Wuhan variant with D614G and M153T mutations in the S protein), (ii) hCoV-19/Kazakhstan/KazNAU-NSCEDI-Kaissar/2021 (Alpha variant, GISAID accession no. EPI_ISL_2349552) with D614G, D1118H, H69del, N501Y, P681H, S982A, T716I, V70del, Y144del mutations in the S protein, and (iii) hCoV-19/Kazakhstan/KazNARU-NSCEDI-5526/2021 (Delta variant, GISAID accession no. EPI_ISL_4198501) with D614G, D950N, E156G, F157del, G142D, H1083Y, L452R, P681R, R158del, T19R, T478K mutations in the S protein were kindly provided by the Masgut Aikimbayev National Scientific Center for Especially Dangerous Infections (NSCEDI) (Almaty, Kazakhstan). Viral titres of SARS-CoV-2 for the Wuhan, Alpha, and Delta variants in the Vero E6 cell line were 6.20 ± 0.00 log_10_ TCID_50_/mL, 5.32 ± 0.13 log_10_ TCID_50_/mL, and 6.45 ± 0.16 log_10_ TCID_50_/mL, respectively. The inactivation of the virus was performed in a BSL-3 laboratory based on an ATCC protocol by heating to 65 °C for 30 min. The completeness of virus inactivation was confirmed by two consecutive passages in the Vero E6 cell culture. All the above-mentioned steps were conducted at the NSCEDI. Further use of inactivated SARS-CoV-2 variants at Nazarbayev University was approved by the biological and chemical safety committee (42/04042022, 25 May 2022).

### 3.3. Instrumentation and Electrochemical Impedance Measurements

EIS measurements were recorded using a PalmSens3 impedance analyzer (PalmSens BV, Houten, The Netherlands) and PSTrace 5.8 software connected to a portable computer and an IDE. EIS measurements were performed within the frequency range between 0.1 Hz and 50,000 Hz with 56 frequencies and 10 mV a.c. All the electrodes were measured in a 0.01 M PBS buffer (pH 7.4) containing 2 mM ferro/ferricyanide [Fe(CN)_6_]^3−^/^4−^ (redox couple buffer) at room temperature inside a custom-made Faraday cage. The signals were recorded in a Nyquist plot, and the circuit fitting was performed using the EIS spectrum analyzer and PSTrace software by fitting the model to the Randles equivalent circuit. The percentage in charge transfer resistance was calculated using the formula [(*R_conc-n_ − R_PBS_*)/*R_PBS_*) × 100] for each concentration according to the *R*_ct_ values obtained with the fitting error value below 5%.

### 3.4. Electrode Surface Cleaning

The surface of the IDE was carefully cleaned by rinsing with 96% ethanol and exposed to an UV/Ozone (Bioforce Nanosciences, Ames, IA, USA) for 20 min. Next, the IDE was rinsed with ethanol and allowed to air-dry, followed by EIS signal measurement after the cleaning in redox couple buffer.

### 3.5. IDE Surface Functionalization

CoV2-RBD-1C aptamer (100 µM) with modifications, as specified in Section 3.1, was combined with a reducing buffer (TCEP) at a 1:2 volume ratio for 1 h. The solution was then diluted with 0.01 M PBS (pH 7.4) to give a concentration of 1 µM aptamer [56]. Before the immobilization, the aptamer solution was heated for 5 min at 95 °C, put on ice for 10 min, and allowed to cool to room temperature. Finally, the IDE was immersed in an Eppendorf tube with a 500 µL aptamer solution and incubated for 4 h at room temperature. After incubation with the aptamer, the IDE surface was rinsed with 0.01 M PBS (pH 7.4). Following the signal measurement after aptamer incubation, the IDE was incubated with 500 µL of 3 mM MCH diluted in 0.01 M PBS (pH 7.4) for 16 h at 4 °C. Stock MCH solution was prepared with absolute ethanol to obtain the 10 mM concentration and stored at −20 °C until use.

### 3.6. Target Detection

Electrochemical measurements were performed on different concentrations of the recombinant SARS-CoV-2 (2019-nCoV) S glycoprotein (RBD) (0.1 pg/mL to 12.8 pg/mL) diluted in 0.01 M PBS (pH 7.4). A 50 µL sample of each concentration of the target protein was incubated on the aptamer functionalized IDE surface for 20 min at room temperature. After the incubation, IDE was rinsed with 0.01 M PBS (pH 7.4) to remove any unbound protein residues from the surface and the signal was measured in the redox couple buffer diluted in 0.01 M PBS (pH 7.4).

To detect the virus, three heat-inactivated SARS-CoV-2 variants, Wuhan, Alpha, and Delta, were serially diluted in 0.01 M PBS (pH 7.4) and were incubated on the IDE surface according to the protocol that was described above for the protein detection.

### 3.7. Specificity Study

Following the IDE surface functionalization, the electrodes were incubated with MERS-CoV Spike/RBD protein fragment, HSA, and influenza HA peptide at the concentration of 0.6 pg/mL diluted in 0.01 M PBS (pH 7.4). The same experimental conditions were followed as described in Section 3.6.

### 3.8. Detection of the Inactivated SARS-CoV-2 Delta Variant in Human Nasal Fluid

The nasal fluid was diluted in 0.01 M PBS (pH 7.4) at the ratio of 1:1000 [76], and the different concentrations of the inactivated SARS-CoV-2 Delta variant (6.45 ± 0.16 × 10^1^–10^5^ TCID_50_/mL) were spiked in the nasal fluid. Next, 50 μL of each concentration was applied to the surface of the aptamer functionalized IDE and incubated for 20 min as described in Section 3.5. All the electrodes were rinsed before all measurements and the signals were measured in the redox couple buffer. All measurements were carried out at room temperature in triplicate, and the mean value of replicates, standard deviations, and standard errors from the mean were used to report the results.

### 3.9. IDE Surface Characterization

Atomic-Force Microscopy (AFM) imaging was performed using the Smart SPM 1000 (AIST-NT, Moscow, Russia). Sample scanning was carried out by using AC-mode (tapping mode). Measurements were made at room temperature. High-resolution AFM images were performed using NSG30_SS cantilevers (TipsNano) with a tip radius curvature of 2 nm, force constant 22–100 N/m, and resonance frequency of 240–440 kHz. AFM image processing was performed using the Gwyddion 2.60 software (Brno, Czech Republic) [77]. The AFM surface scanning was performed after each IDE surface modification step and target capturing including a viral particle on the IDE surface as described in Section 3.4, Section 3.5 and Section 3.6: (i) the bare IDE, (ii) an aptamer immobilized IDE surface after a 4 h of incubation, (iii) an MCH backfilled IDE surface after 16 h of incubation, (iv) an IDE incubated with a 0.6 pg/mL of SARS-CoV-2 S glycoprotein, and (v) an IDE surface capturing an inactivated SARS-CoV-2 Delta variant. After each incubation step, the IDE was carefully rinsed with dH_2_O and allowed to air-dry. AFM imaging on the mica surface was performed by applying a 2 μL sample of 10^3^ TCID_50_/mL of an inactivated SARS-CoV-2 Delta variant on the mica sheet and allowing it to air-dry.

### 3.10. Data Analysis

EIS data was analyzed using the PSTrace 5.8 software (PalmSens BV, Houten, The Netherlands) and EIS Spectrum analyzer 1.0 free software (Minsk, Belarus) [78]. All the impedance spectra data were obtained by conducting three individual measurements. The values obtained from equivalent circuit fitting were presented as mean ± standard errors (SEM) and standard deviation (SD). All the experiments were performed in triplicate. Data analysis and graph plotting were carried out using OriginLab v.2016 (OriginLab Corporation, Northampton, MA, USA) and GraphPad Prism v.9.1.0 for Windows (GraphPad Software, San Diego, CA, USA). Statistical significance was analyzed using analysis of variance (ANOVA), and the significance level was set as *p* < 0.05. LOD was calculated from the calibration curve.

## 4. Conclusions

Herein, we presented the results of the development and application of an EIS aptasensor for the detection of three variants of SARS-CoV-2 (Alpha, Delta, and Wuhan) based on the recognition of the S glycoprotein. The aptasensor is based on a simple immobilization technique and surface chemistry (co-immobilization of MCH) on and IDE surface that provides a better orientation for the aptamer, hence, greater accessibility to the target protein. Successful functionalization of the IDE surface with the 18-HEG modified aptamer allowed us to obtain excellent sensitivity towards the RBD of the SARS-CoV-2 S glycoprotein. We achieved an LOD of 0.4 pg/mL with 20 min of incubation time and 2.5 min for EIS detection time, showing the potential of the aptasensor as a diagnostic tool for COVID-19. In addition, the resulting aptasensor exhibited high specificity to the target SARS-CoV-2 S glycoprotein and little or no response toward the non-target ones. The surface characterization using AFM confirmed the immobilization of the aptamer on the IDE surface and capture of the SARS-CoV-2 S glycoprotein and the inactivated Delta variant. The developed aptasensor was also evaluated through the detection of three heat-inactivated SARS-CoV-2 variants in a buffer with the detection limits of 6.45 ± 0.16 × 10^3^ TCID_50_/mL (Delta), 5.32 ± 0.13 × 10^2^ TCID_50_/mL (Alpha), and 6.20 × 10^4^ TCID_50_/mL (Wuhan). Moreover, the prospective practical application of the developed aptasensor was assessed by detecting the heat-inactivated Delta variant in human nasal fluid, with a resulting sensitivity of 6.45 ± 0.16 × 10^3^ TCID_50_/mL. Taken together, these results suggest the promising potential of the current aptasensor considering its fast, sensitive, and specific performance. Future studies should validate the analytical performance of the aptasensor in clinical samples.

## Data Availability

Not applicable.

## Supplementary Materials

The following supporting information can be downloaded at: https://www.mdpi.com/article/10.3390/ijms232113138/s1, Table S1. Infectious activity titer of SARS-CoV-2 virus isolates grown in Vero E6 cell culture.; Table S2. Cytopathic effect evaluation of SARS-CoV-2 virus isolates.; Table S3. Cycle threshold (Ct) values from real-time RT-PCR.; Figure S1. TEM micrographs of the inactivated SARS-CoV-2 Delta variant with a magnification of (a) 50 nm and (b) 100 nm.
