# Supplementary material for: Sensitive Detection of SARS-CoV-2 Variants Using an Electrochemical Impedance Spectroscopy Based Aptasensor
Source: Int J Mol Sci. 2022 Oct 28;23(21):13138. doi: 10.3390/ijms232113138 (PMC9656073; doi:10.3390/ijms232113138)
Supplement: Supplementary file 1 [file ijms-23-13138-s001.zip › ijms-1981185-supplementary.pdf]

International Journal of
Molecular Sciences

Supplementary information

Sensitive Detection of SARS-CoV-2 Variants Using an Electrochemical Impedance Spectroscopy Based Aptasensor

Assem Kurmangali [1], Kanat Dukenbayev [2,†] and Damira Kanayeva [1,*]

[1] Department of Biology, School of Sciences and Humanities, Nazarbayev University, Astana 010000, Kazakhstan
[2] Department of Electrical and Computer Engineering, School of Engineering and Digital Sciences, Nazarbayev University, Astana 010000, Kazakhstan
* Correspondence: dkanayeva@nu.edu.kz
† Current employment address: King Abdullah University of Science and Technology, Thuwal 23955, Saudi Arabia.

Methods

Virus propagation and determination of cytopathic effect

Vero E6 cell lines (ATCC® CRL1586™) were grown in Dulbecco's Modified Eagle's medium (DMEM) supplemented with 10% fetal bovine serum (FBS). The day before the propagation with the virus, cells were seeded in the quantity of 20,000 cell into 96 well-plates. 10-fold dilutions of SARS-CoV-2 (Wuhan, Alpha, Delta variants) were prepared by adding a 30 µl of virus suspension to the 270 µL of DMEM medium supplemented with 2% FBS for the determination of cytopathic effect (CPE). DMEM media with 2% FBS without the virus was used as a negative control. After reaching the confluency of 95 – 100% of the Vero E6 cells, the growth medium was removed and the 200 µL of each dilution of the virus was added to 96 well-plates. After 1 h, the virus containing medium was removed from each well and fresh DMEM with 2% FBS was added to the wells. Infected cells were incubated for 5 days at 37 °C with 5% CO_2 until the presence of the CPE. The 50% tissue culture infectious dose ($TCID_{50}$) was recorded and calculated by Reed-Muench method.

Validation of the SARS-CoV-2 presence was performed by reverse transcriptase polymerase chain reaction in real time. RNA extraction was performed using a commercial GeneJET Viral DNA/RNA Purification Kit (Thermo Scientific, Cat # K0821) according to the manufacturer's instructions.

All the work with SARS-CoV-2 virus propagation and inactivation was performed in accordance with the International Standard ISO 35001:2019 «Biorisk management for laboratories and other related organizations» in BSL-3 laboratory at the Masgut Aikimbayev National Scientific Center for Especially Dangerous Infections (Almaty, Kazakhstan).

Transmission electron microscopy

Heat inactivated SARS-CoV-2 Delta variant sample was fixed in 2.5% glutaraldehyde (cat.no. G5882, Sigma-Aldrich) in 1:1 ratio overnight at 4°C. A drop of the fixed sample was applied on a carbon grid and allowed to dry, then, carefully washed twice with 0.1 M PBS (pH 7.4) for 10 min. Post-fixation was performed with 1% osmium tetroxide (cat.no. 75632, Sigma-Aldrich) for 1 h, followed by washing in PBS for 10 min twice. Samples were then dehydrated through the series of increasing ethanol concentrations for 10 min (30, 50, 70, 80, and 96%). Imaging was performed on a Jeol JEM - 1400 Plus transmission electron microscope (Japan Electron Optics Laboratory Co., Ltd).

Figures and tables

Table S1. Infectious activity titer of SARS-CoV-2 virus isolates grown in Vero E6 cell culture.

**Virus isolate/ Control**	**Virus dilutions / CPE**								**Virus titer, $\log_{10}$ $TCID_{50}$/mL**
	**10^{-1}**	**10^{-2}**	**10^{-3}**	**10^{-4}**	**10^{-5}**	**10^{-6}**	**10^{-7}**	**10^{-8}**	
Wuhan variant	++++<br>++++	++++<br>++++	++++<br>++++	++++<br>++++	++++<br>++++	----<br>----	----<br>----	----<br>----	6.20 ± 0.00
Alpha variant	++++<br>++++	++++<br>++++	++++<br>++++	++++<br>++++	--+-<br>----	----<br>----	----<br>----	----<br>----	5.32 ± 0.13
Delta variant	++++<br>++++	++++<br>++++	++++<br>++++	++++<br>++++	++++<br>++++	-+-+<br>----	----<br>----	----<br>----	6.45 ± 0.16
Negative control	--------								
Positive control	++++++++								

* "+" presence of CPE; "-" absence of CPE; "Negative control" - media without the virus; "Positive control" - non-diluted virus in media.

Table S2. Cytopathic effect evaluation of SARS-CoV-2 virus isolates.

**Dilution**	**Wuhan ($TCID_{50}$/mL)**	**Alpha ($TCID_{50}$/mL)**	**Delta ($TCID_{50}$/mL)**
10^{-1}	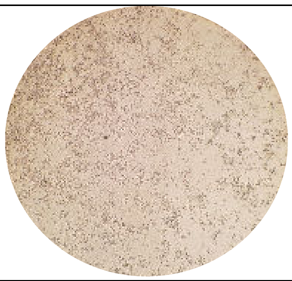<br>(158 489)	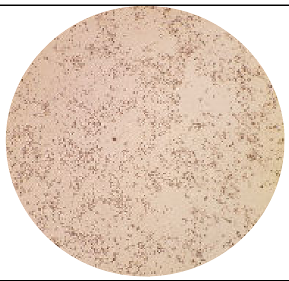<br>(20 892)	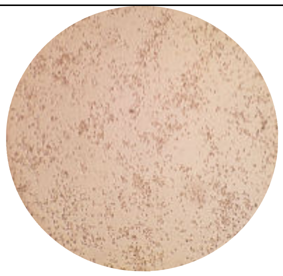<br>(281 838)
10^{-2}	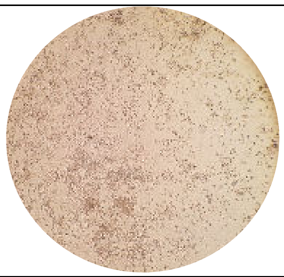<br>(15 848)	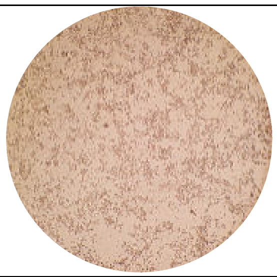<br>(2 089)	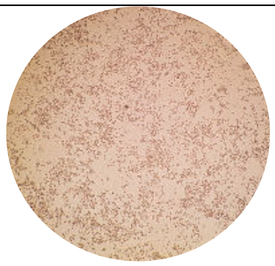<br>(28 183)

10^{-3}	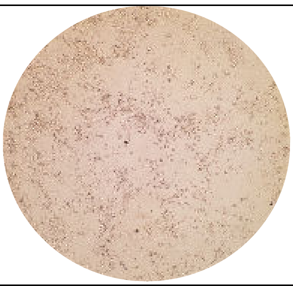	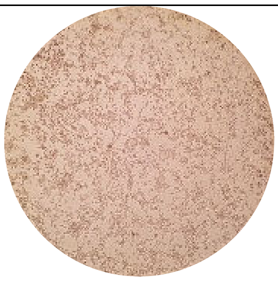	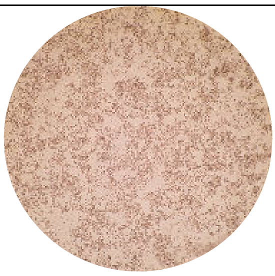
	(1 584)	(208)	(2 818)
10^{-4}	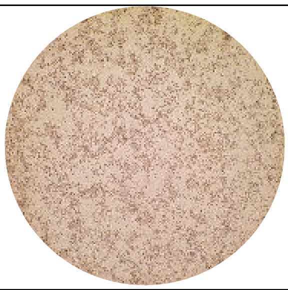	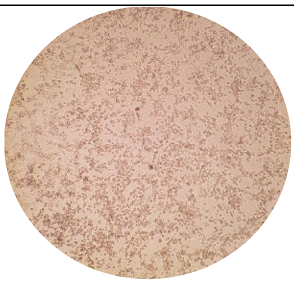	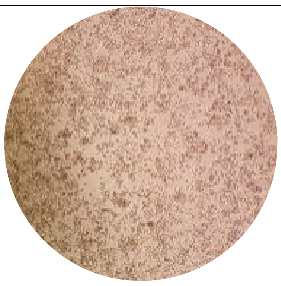
	(158)	(20)	(281)
10^{-5}	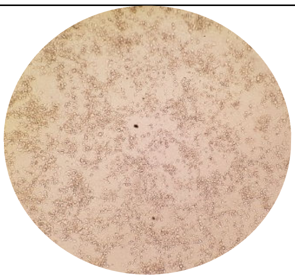	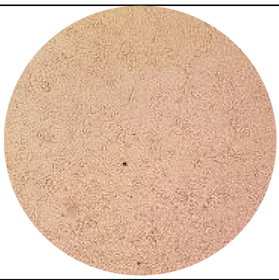	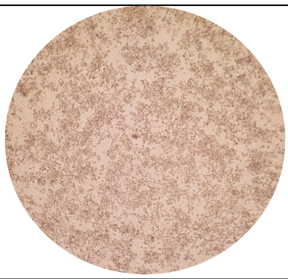
	(15)	(2)	(28)
10^{-6}	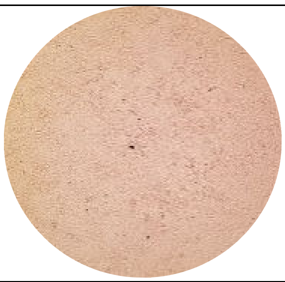	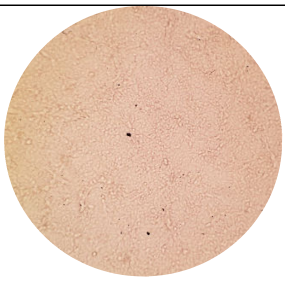	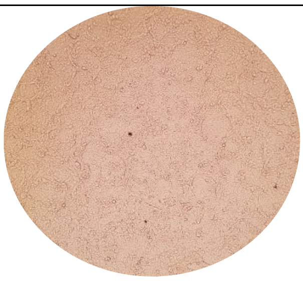
	(1,5)	(0)	(2,8)
Negative control	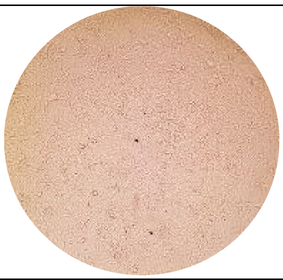	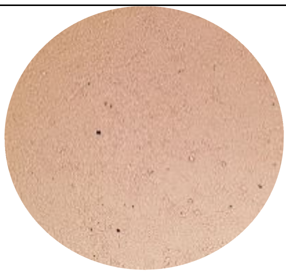	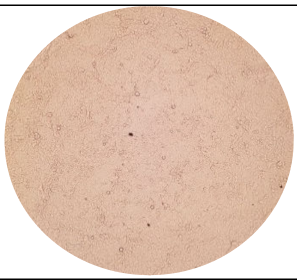
	(0)	(0)	(0)

Positive control	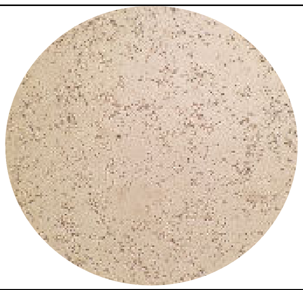	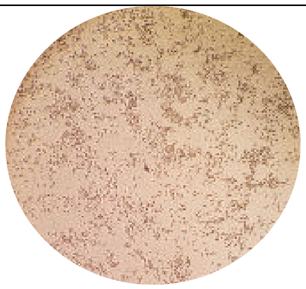	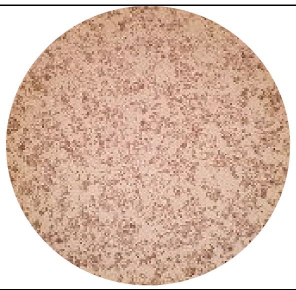
	(1 584 893)	(208 929)	(2 818 382)

Table S3. Cycle threshold (Ct) values from real-time RT-PCR.

**Virus isolate/Control**	**Ct / dilutions ($TCID_{50}$/mL)**						
	**10^{-1}**	**10^{-2}**	**10^{-3}**	**10^{-4}**	**10^{-5}**	**10^{-6}**	**10^{-7}**
Wuhan variant	15.08 (158489)	18.29 (15848)	21.83 (1584)	25.23 (158)	29.12 (15)	33.61 (1.5)	- (0)
Alpha variant	16.55 (20892)	20.66 (2089)	25.18 (208)	28.57 (20)	33.12 (2)	- (0)	- (0)
Delta variant	14.58 (281838)	18.75 (28183)	22.34 (2818)	25.86 (281)	29.34 (28)	33.63 (2,8)	- (0)
Negative control	-						
Positive control	19.83						

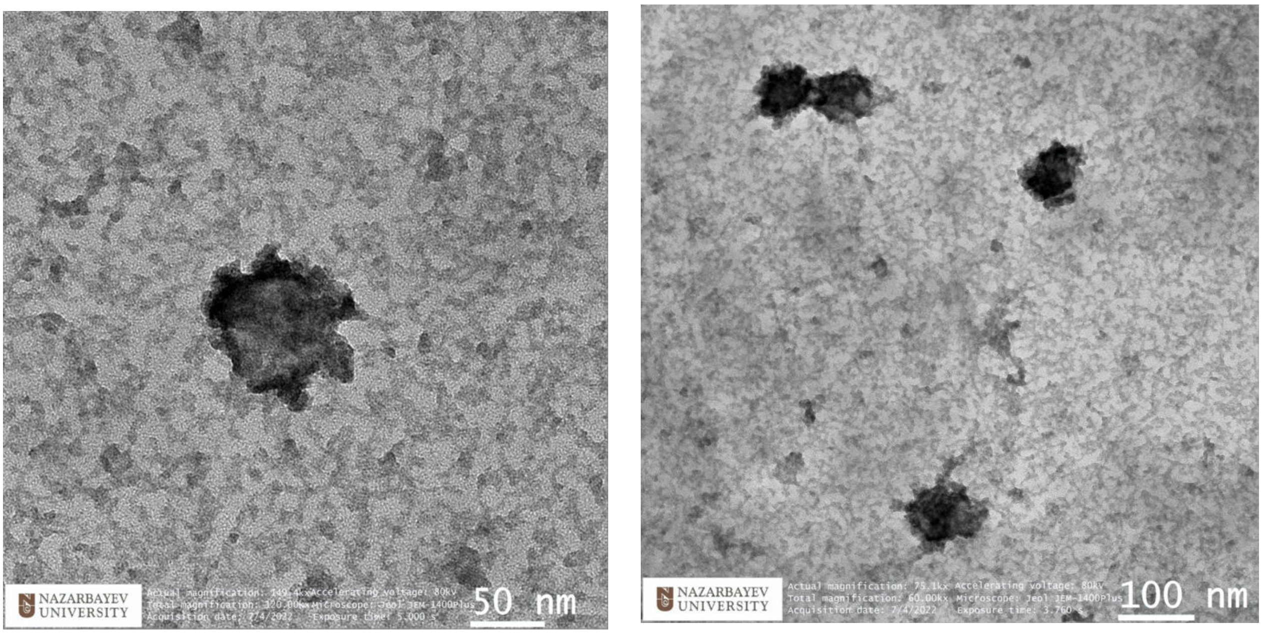

(**a**) (**b**)

Figure S1. TEM micrographs of the inactivated SARS-CoV-2 Delta variant with a magnification of (**a**) 50 nm and (**b**) 100 nm.
